# Biomarkers in the Diagnosis and Prediction of Medication Response in Depression and the Role of Nutraceuticals

**DOI:** 10.3390/ijms25147992

**Published:** 2024-07-22

**Authors:** Cristina Beer, Fiona Rae, Annalese Semmler, Joanne Voisey

**Affiliations:** 1 Centre for Genomics and Personalised Health, School of Biomedical Sciences, Faculty of Health, Queensland University of Technology, Kelvin Grove, QLD 4059, Australia; cristina.beer@hdr.qut.edu.au (C.B.); f.rae@qut.edu.au (F.R.); 2 School of Clinical Sciences, Faculty of Health, Queensland University of Technology, Kelvin Grove, QLD 4059, Australia; annalese.semmler@qut.edu.au

**Keywords:** depression, biomarkers, pharmacogenomics, nutraceuticals

## Abstract

Depression continues to be a significant and growing public health concern. In clinical practice, it involves a clinical diagnosis. There is currently no defined or agreed upon biomarker/s for depression that can be readily tested. A biomarker is defined as a biological indicator of normal physiological processes, pathogenic processes, or pharmacological responses to a therapeutic intervention that can be objectively measured and evaluated. Thus, as there is no such marker for depression, there is no objective measure of depression in clinical practice. The discovery of such a biomarker/s would greatly assist clinical practice and potentially lead to an earlier diagnosis of depression and therefore treatment. A biomarker for depression may also assist in determining response to medication. This is of particular importance as not all patients prescribed with medication will respond, which is referred to as medication resistance. The advent of pharmacogenomics in recent years holds promise to target treatment in depression, particularly in cases of medication resistance. The role of pharmacogenomics in routine depression management within clinical practice remains to be fully established. Equally so, the use of pharmaceutical grade nutrients known as nutraceuticals in the treatment of depression in the clinical practice setting is largely unknown, albeit frequently self-prescribed by patients. Whether nutraceuticals have a role in not only depression treatment but also in potentially modifying the biomarkers of depression has yet to be proven. The aim of this review is to highlight the potential biomarkers for the diagnosis, prediction, and medication response of depression.

## 1. Introduction

Around 3.8% of the global population, which includes approximately 5% of adults (with rates at 4% for men and 6% for women), along with 5.7% of adults aged over 60 years, are estimated to experience depression [1]. This means that around 280 million people worldwide are affected by depression [1]. The COVID-19 pandemic greatly exacerbated this rate, with data showing that there were an additional 53.2 million cases of major depressive disorder diagnosed globally in 2020, which is an increase of 27.6% [2]. The World Health Organization (WHO) projections estimate that major depression will be placed as the leading cause of disability by 2030 [3]. 

Most patients with depression will present at some stage to their general practitioner [4]. Unfortunately, the diagnosis of depression is not always an easy one to make, especially where there are comorbidities obscuring the clinical picture, or where symptoms are underreported [5]. The latter is particularly true in cases of mild depression, which is missed in around half of such patients in the general practice setting [6]. This group is likely to experience persisting or relapsing symptoms of depression [7,8]. 

The challenge in diagnosing depression stems from its predominantly clinical nature, often necessitating the assessment of symptoms through checklist-based approaches, of which there are several options available [9]. This especially poses a challenge in mild depression, as it often meets fewer of the diagnostic criteria for major depression [10]. This lack of objectivity not only makes it difficult for diagnosis but can also pose a barrier to early and targeted treatment.

## 2. Current Treatment of Depression

The mainstay of depression treatment is psychological counselling and anti-depressant medication, with the latter often reserved for cases of moderate-to-severe depression [11]. The last few decades have seen a surge in the use of antidepressants worldwide [12]. There is debate as to whether this surge corresponds to overprescribing or is fuelled by the increased incidence in depression [13,14]. More commonly, depression often goes undiagnosed, particularly in the elderly, and is therefore undertreated [15,16]. Conversely, older adults with depression are at significant risk of polypharmacy, which increases morbidity and mortality in this age group [17,18]. As with all people with depression, this highlights the importance of diagnosing and managing depression appropriately.

Although progress has been made since the STAR*D (Sequenced Treatment Alternatives to Relieve Depression) report [19], a landmark study undertaken in 2004 aimed at evaluating the effectiveness of various treatment strategies for individuals with major depression, work still needs to be carried out. The issue with prescribing anti-depressant medications lies in the variable patient response (benefit to medication) rate. Around 30% of patients will be complete non-responders that is they will not experience any therapeutic benefit from the medication [20]. A patient who responds to treatment should achieve a 50% or greater reduction in depressive symptoms as measured by a standardized rating scale. A patient who does not achieve a meaningful reduction in depressive symptoms is typically defined as having less than a 25% reduction in symptom severity. Furthermore, up to two-thirds of patients with depression will not respond to the first anti-depressant they trial and may take up to trialling two or three agents to find one that works for them [21]. Additionally, greater than a third of individuals who trial antidepressants will report an adverse reaction, further leading to reduced medication adherence [22]. Of those that fail to respond to medication, known as medication-resistant, around 30% have attempted suicide at least once [23]. An Asia-Pacific expert consensus has, in recent years, defined medication resistance as a failure to achieve remission (<50% improvement in depressive symptoms or inability to return to work/study) after trialling at least two antidepressants from the same or different classes, at a minimum effective antidepressant dose for 6–8 weeks [19]. The estimated total disease burden of medication-resistant depression, measured in Disability Adjusted Life Years (DALYs), is high worldwide, and the combined annual direct (medical appointments/medications/treatments/hospitalisations) and indirect costs (lost productivity/absenteeism) is frequently reported to be billions of dollars [24,25].

Selecting the appropriate antidepressant for an individual patient is not a straight-forward clinical decision. The standard of care of depression management has limited supportive evidence and is based on subjective factors such as baseline symptoms, potential side effects, patient preference, and cost [26,27]. In most cases, antidepressants must be taken for weeks before a response can be assessed. Furthermore, the ensuing multiple medication trial for most patients with depression can take weeks to months and risks disillusionment and attrition to care [28]. 

Many of the pharmacological recommendations outlined in the STAR*D report, which were centred around the sequential use of antidepressant medications (and/or the addition of an augmenting medication, such as mood stabilisers) until a clinical response was achieved, are still undertaken today [19]. This ad hoc approach where multiple medication steps are often necessary to achieve remission of major depression is not optimal. The STAR*D study did highlight the importance of considering individual characteristics including genetics and other biological markers or ‘biomarkers’, as well as patient preferences, and, in doing so, offered an insight into the future of depression management, which is towards personalised medicine as a more targeted and effective treatment approach [19]. Nearly two decades later, pharmacogenomics and precision medicine are still not widely used in Australia. This contrasts with other countries, such as the UK and Netherlands, which are at the forefront of integrating pharmacogenomics into clinical practice. Both countries have implemented pharmacogenomic testing programs into clinical practice to guide medication prescribing and have incorporated pharmacogenomic information into their clinical practice guidelines [29,30].

**Figure 1:**
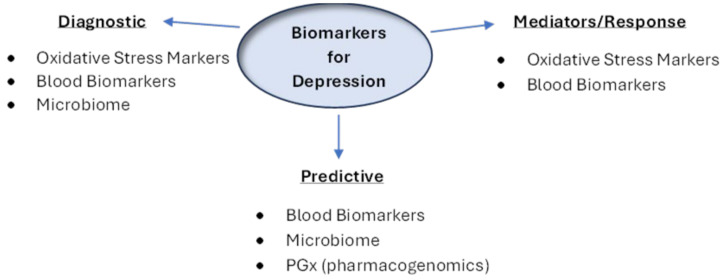
Biomarkers for depression. Biomarkers identified before treatment initiation are classified as diagnostic or predictive. Diagnostic markers identify a patient with depression and predictive markers determine overall likelihood of response to medication. Mediators are biomarkers used after medication initiation and help predict overall likelihood of response/remission.

Broadly, the factors suggested to impact variable response to medication can be classed as extrinsic or intrinsic. Extrinsic factors include issues of adherence (to medication taking), nutrient intake, as well as concomitant alcohol and drug use [31]. These factors will not be extensively discussed here but should be considered potential confounders. Intrinsic factors to the patient, such as biomarkers, provide promise to objective depression diagnosis and more targeted treatment, and they include genetic markers, markers of oxidative stress and inflammation, as well as other biochemical factors that are able to be readily detected in blood samples. In addition, there is growing research into the impact of the gut microbiome in medication response (Figure 1). 

There is a definite need for a more targeted, personalised approach to depression treatment and, as this is likely to involve many factors, keeping a multifaceted, ‘bigger picture’ approach in mind will be essential. This area has yet to be explored, thus this paper presents a novel strategy for informing the treatment of depression in clinical practice.

## 3. Potential Role of Pharmacogenomics (PGx) in Predicting Response to Depression Medication

Of the intrinsic factors potentially affecting medication response, only genetic influence has been researched widely [32,33,34,35,36] The advent of genomic testing has made this a possibility, and the science of combining pharmacology with genomics or ‘pharmacogenomics (PGx)’ potentially offers an avenue for more targeted medication in depression by minimising the trial-and-error approach to prescribing antidepressants [36,37]. Identifying genetic variations, such as single nucleotide polymorphisms (SNPs) or copy number variations (CNVs), that could potentially lead to changes in pharmacokinetic or pharmacodynamic drug response may provide an opportunity to predict the way in which an individual responds to drugs [38,39].

In depression, antidepressants are a common treatment choice. However, not all patients respond to these drugs [40]. It is predicated that genetic variation may account for up to 42% of the variation in individual antidepressant response rates [41]. Variation in specific pharmacogenes encoding hepatic cytochrome P450 drug-metabolising enzymes (CYP2D6, CYP2C19, CYP1A2, CYP3A4, and CYP2B6), transport carriers (solute carrier 6A2 (SLC6A2), solute carrier 6A4 (SLC6A4), adenosine triphosphate binding cassette 1 (ATP-B1), and drug receptors (serotonin receptor, HTR2A) could influence the effectiveness and tolerability of antidepressant medications [37,42,43,44]. 

### 3.1. Pharmacokinetic Gene Targets

Of all the potential pharmacogenomic markers associated with response to antidepressants, those genes encoding the CYP450 drug metabolising enzymes have been widely studied and their use in antidepressant prescribing has been validated, specifically *CYP2D6*, *CYP2C19*, *CYP3A4*, *CYP1A2*, and *CYP2B6* [45]. It is important to note that approximately 85% of antidepressants are metabolised by CYP2D6, 38% by CYP2C19 and CYP3A4, and 5% by CYP2B6 [46]. Genetic variations in these enzymes, both in sequence and copy number, can result in altered drug metabolism, leading to variations in drug efficacy and side effect profiles [47]. 

The altered metabolic activity of CYP450 enzymes due to genetic variation means individuals are phenotypically poor, intermediate, extensive, rapid and ultrarapid metabolisers of antidepressant medications, which may affect their drug plasma concentrations. For example, individuals who are poor metabolisers of CYP2D6 may experience increased drug concentrations and could be at higher risk of adverse effects when treated with certain antidepressant medications metabolised primarily by this enzyme. On the other hand, ultrarapid metabolisers of CYP2D6 may require higher doses to achieve therapeutic concentrations. The CYP450 enzymes and commonly prescribed antidepressant medications they metabolise are listed in Table 1.

Furthermore, it is important to note that CYP450 genetic variation is impacted by ethnicity. For example, approximately 7–10% of Caucasians are poor CYP2D6 metabolisers while 19% African Americans are ultra rapid CYP2D6 metabolisers of psychiatric medications, and these may be associated with a reduced response to medication [50,51]. This phenomenon is believed to be attributed to single nucleotide polymorphisms (SNPs) in *CYP450* genes or whole gene deletions or duplications of *CYP2D6* [52,53,54]. Understanding these genetic differences could allow for testing to be tailored to the ethnic groups that would receive the most benefit.

### 3.2. Pharmacodynamic Gene Targets

For the most prescribed class of medication for depression, the selective serotonin reuptake inhibitors (SSRIs), several gene variations have been implicated in the response to these medications [55]. For instance, variations in the sequence of the promoter region of the *SLC6A4* gene, which encodes a serotonin transporter that is responsible for serotonin reuptake into the presynaptic neuron, can alter serotonin levels and lead to either a decreased or increased response to SSRIs [45]. 

Testing for methylenetetrahydrofolate reductase (*MTHFR*) gene variants has also been a point of interest in mental health research [56,57,58]. *MTHFR* is a key regulatory enzyme in folate and homocysteine metabolism and polymorphisms in this gene occur in 60–70% of the population [58], resulting in a reduced ability to utilise folate in its non-activated or ‘methyl’ form [59]. There is a suggested link between abnormal folate metabolism and high levels of homocysteine in increased incidence for depression and reduced antidepressant effectiveness [59]. In a study on SSRI-resistant major depressive disorder, 148 patients were randomly assigned to receive L-methylfolate, placebo followed by L-methylfolate, or placebo for 60 days [60]. In a second, similar trial with 75 patients, L-methylfolate was given at 15 mg/day. The first trial showed no significant differences in outcomes, while the second trial found that adjunctive L-methylfolate at 15 mg/day significantly improved response rates and symptom severity compared to SSRI plus placebo, with good tolerance and no increase in adverse events. Thus, adjunctive L-methylfolate at 15 mg/day appears to be an effective and safe medication for SSRI-resistant depression. The same authors conducted a 12-week open clinical trial of fluoxetine on 49 participants with MDD, finding that the C677T and A2756G polymorphisms did not significantly impact antidepressant response [61]. These two studies suggest that while the C677T and A2756G polymorphisms do not significantly influence antidepressant response in MDD patients, adjunctive L-methylfolate can be an effective and safe treatment for patients with SSRI-resistant depression, improving response rates and symptom severity without increasing adverse events.

### 3.3. Clinical Utility of Pharmacogenomics

Worldwide collaboratives exist to evaluate and report the interplay between various SNPs and medications, determining the potential clinical relevancy of pharmacogenes and suggesting if and how this information can be applied in clinical practice. Of these, the Clinical Pharmacogenetics Implementation Consortium (CPIC) and the Dutch Pharmacogenetics Working Group (DWPG) are the most frequently described [62,63]. The evaluated data are made available through publications and through public-accessible knowledgebase platforms like PharmGKB [64,65]. These consortiums also provide clinical recommendations when prescribing antidepressant medications [66,67]. It is important to note that recommendations may not be available for all medications as the consortiums are limited by the quality and quantity of the pharmacogenomics studies available. As more research on the different pharmacogenes is made available, these recommendations are likely to expand so we need to consider this an evolving field.

Cost has also been a cited barrier for the implementation of pharmacogenomics into clinical practice. The accessibility today of commercially available PGx testing makes use in clinical practice a reality and it has been shown to be relatively cost-effective. A report in 2008 estimated that the widespread implementation of PGx testing in Australia could yield savings in excess of $1 billion annually by the avoidance of adverse medication reactions alone [68] Similar reports have been published internationally [69]. However, whilst these reports compare a myriad of costs including that of pharmacogenomic testing with the cost of treatment failure, cost to the healthcare sector, cost to the individual (e.g., prescription costs, time off work), and have concluded that pharmacogenomics has the potential to be cost-effective, the data need to be approached cautiously with consideration to geographical context (healthcare sectors are not equivalent globally) as well as reference to other factors that may improve clinical outcome (e.g., non-pharmacological interventions) [70].

The evidence supporting the clinical effectiveness of pharmacogenomic screening for depression has been conflicted. Whilst numerous clinical trials have indicated a potential benefit for utilising pharmacogenomic-guided testing to inform the selection of appropriate antidepressant therapy [71,72,73], there have been alternative studies where the clinical efficacy of pharmacogenomic-guided antidepressant therapy has been unclear [35,74,75,76]. The design of the clinical trials evaluating pharmacogenomic-guided therapy has been raised as a potential contributor to the variation in trial outcomes [77,78]. Furthermore, a rapid review and meta-analysis published in 2023, which evaluated 10 RCTs (n = 4333) reported a similar outcome [78]. Whilst pharmacogenomics is a promising tool, the widescale benefit and practicality of utilising PGx testing for depression in clinical practice, however, has yet to be established. Most pharmacogenomic trials to date have had limited ethnic and gender diversity, with most participants being female with a European background [77] Designing trials to encompass gender and ethnic diversity would be essential if this technology is to be translated into clinical practice. Determining the impact of pharmacogenomic-guided therapy on symptom score has also been highlighted as an important outcome as not all patients achieve remission [78]. Furthermore, variation and lack of transparency between the type of tests used across the different trials and the potential impact that this may have on outcomes highlight a need for standardised pharmacogenomic testing [77]. Pharmacogenomic-guided testing has the potential to aid in antidepressant selection for the management of patients with MDD, but addressing these limitations is needed before its integration into clinical practice.

## 4. Role of Oxidative Stress Markers in Depression Diagnosis and Treatment 

Oxidative stress has been strongly causally linked to depression, with elevations in reactive oxygen species (ROS) and reduced oxidative defence mechanisms being observed [79,80]. This aberration in redox status whereby the normal cellular metabolites of oxidative metabolism, namely oxygen and hydrogen peroxide, fail to be counterbalanced and controlled by endogenous antioxidant defence mechanisms, such as superoxide dismutase (SOD), catalase, and glutathione peroxidase, leads to free radical attack on proteins, DNA, and lipids [81]. As the brain has relatively low levels of antioxidant defences, as well as a high lipid content, it makes it highly susceptible to attack by ROS [81]. The ensuing neuroinflammation from oxidative stress in depression is difficult to measure in clinical practice.

The urinary pyrrole test, which measures hydroxyhemepyrrolin-2-one (HPL) in urine, may be a marker of oxidative stress as suggested in our recent publication [82]. The HPL detected in urine has been shown to originate from the fragmentation of regulatory haem by ROS [82]. This occurs due to excess production of peroxides from excess physical and/or physiological stress. Elevated levels of urinary pyrroles have been clinically linked to a range of psychiatric conditions, including depression [83,84,85,86]. Whether the use of urinary pyrrole testing in clinical practice can act as a biomarker for depression and response to treatment requires further validation. 

Many of the changes in oxidative status may be directly related to increased inflammatory response and may be due to the presence of other systemic inflammatory illnesses, such as endocrine and metabolic disorders as well as cardiovascular disorders and need to be accounted for in any interpretation of study results.

## 5. Role of Blood Biochemistry Markers in Depression Diagnosis and Treatment 

Of the potential blood biomarkers linked with depression, the C-reactive protein (CRP) is the most investigated to date [87,88,89,90]. As CRP is a measure of inflammation and has been shown to be high in depression, it points to the association among chronic inflammation, oxidative stress, and depression [89,90,91,92]. In fact, CRP may not only be able to indicate the presence of depression, with levels correlating with the severity of symptoms, but may also predict response to medication [93]. Studies have indicated that for patients with a high CRP level, Selective Noradrenaline Reuptake Inhibitors (SNRIs) may be more effective as a medication, whereas those with low CRP levels, SSRIs are more effective [94,95,96]. A study by Halaris et al. [97] investigated the association between *CRP* gene polymorphisms and clinical outcomes in patients receiving psychiatric medication. Carriers of the *CRP* SNPs rs3093059 and rs3093077 had higher baseline CRP levels, while non-carriers receiving celecoxib showed higher response and remission rates and lower stress scores. These findings suggest that determining *CRP* SNP carrier status and measuring pretreatment CRP levels could contribute to personalized psychiatric care, although further replication of these results is needed. It should be noted that as an indicator of inflammation, it has been associated with numerous conditions, not just depression, including infections, cancer, obesity, and autoimmune diseases and so, by itself, lacks the specificity and sensitivity to be a diagnostic (and prognostic) tool in depression. 

In addition, evidence indicates that patients with depression show substantial variations in certain metabolic factors including altered glucose, lipids and albumin profile, as well as abnormal serum leptin and insulin levels [98]. These factors can be easily measured in general practice and point to the bidirectional relationship among metabolic disorders and depression, likely in significant part due to lifestyle factors [99]. When analysing study data to determine if a correlation exists in depression, it is likely that diet and exercise as key components of lifestyle contributors to metabolic disorders need to be accounted for the interpretation of these key markers [100,101]. 

The link between depression and hormonal response is in the domain of neuroendocrinology, and studies have suggested that alterations in cortisol as well as thyroid hormones may be an indicator of depression and medication response in clinical practice [102,103,104]. The hypothalamic–pituitary–adrenal (HPA) axis is overactive in those with depression, likely representing a heightened stress response, and a higher cortisol level has been indicated as marker of poor response to medication in depression [105]. Hypothyroidism, as measured in serum as a suppressed thyroid stimulating hormone (TSH), may play a causal role in depressed mood and the adjunct of a synthetic thyroid hormone, tetraiodothyronine (T4), is sometimes used in clinical practice to augment response to antidepressant medication in recalcitrant cases [105,106,107,108]. Furthermore, thyroid response has been shown to normalise with successful treatment of depression [108]. 

Another novel blood biochemical marker of mental health illness is the zinc/copper ratio. An elevation in copper and a reduction in the zinc/copper ratio has been linked with neuropsychiatric symptoms and cognitive impairment [109,110]. Copper and zinc share a reciprocal relationship, and as a high level of copper has been shown to be neuroinflammatory and zinc shown to be neuroprotective, an aberration in the zinc/copper ratio may be a marker of mental illness [111,112]. This has yet to be validated as a useful indicator of mental health illness in clinical practice. It is, however, a readily available pathology test and may prove useful as part of the diagnostic work-up as well as in gauging response to treatment in depression. 

Recent research has explored the role of iron metabolism in mental disorders [113]. Iron is linked to norepinephrine regulation in the brain, impacting neuroplasticity and the function of the prefrontal cortex and hippocampus. Additionally, iron regulates the brain-derived neurotrophic factor (BDNF) levels, which are crucial for neurotransmitter synthesis and neuronal function. Disruptions in iron bioavailability may affect the BDNF levels and neurotransmitter production, potentially contributing to depression [114]. While the association between glutamatergic system dysfunction and depression is still being investigated, evidence suggests that iron deficiency can influence mood disorders by affecting glutamate production [115]. Studies suggest that anaemia or iron deficiency may contribute to postpartum depression [115].

Given the complex and heterogenous nature of depression, it is unlikely that these blood biochemical markers on their own would be predictive of medication response in depression, but a blood biochemistry panel may provide useful clinical insights.

## 6. Role of Microbiome in Depression Diagnosis and Treatment 

The proposed link between depression and the microbiome is dysfunction in the microbiota–gut–brain (MGB) axis [116,117]. Studies have suggested that relative to healthy controls, those with depression exhibit a reduction in certain organisms (or microbiota), e.g., Bifidobacterium, Lactobacillus, and Firmicutes, and an increase in other bacteria, such as the Bacteroidetes [117,118]. The proposed mechanism of action of the MGB axis is complex and involves immune system activation (through inflammatory mediators), neurotransmitter production (such as serotonin, gamma aminobutyric acid (GABA), and glutamate), and effects exerted through microbiota metabolites (such as short-chain fatty acids (SCFA)) [119]. Furthermore, the enteric nervous system hypothesis suggests a bidirectional neural pathway between the gut and the central nervous system via the Vagus nerve, which is part of the autonomic nervous system [119]. 

Interestingly, the gut microbiome appears to be different in those individuals that respond to medication in depression and in those that do not respond. Those that do not respond to antidepressant medication have been observed to have elevated levels of microbiome tryptophan metabolism, which, in turn, lowers the bioavailability of tryptophan for serotonin synthesis [120]. Whether this is a clinically significant finding is largely unknown. Paradoxically, studies have shown that the microbiome undergoes alterations in its composition in response to anti-depressant medication to resemble that of non-depression controls [121]. Whether this alteration in microbiota composition is responsible for the improvement in depression symptoms per se has yet to be determined. The question arises as to what occurs in microbiome of medication non-responders? Does their microbiome not undergo alterations in response to medication as expected or is the mechanism of their medication non-response not related to their microbiome composition, i.e., are other factors at play? A recent systematic review and meta-analysis explored the bidirectional relationship between psychotropic medications and the gut microbiome, finding that psychotropics can modify gut microbiome composition, while the gut microbiome may impact the efficacy and tolerability of these medications, suggesting the potential for improving treatment strategies in psychiatry through understanding psycho-pharmacomicrobiomics [122].

Preliminary research has suggested that the microbiome may mediate oxidative stress in the host [123]. Depending on the constituents of the microbiome, the level of free radical production varies, and this appears to determine the oxidative stress load [123]. This, in turn, has been observed to impair mitochondrial, cognitive, and neurological function [123,124]. Whether the microbiome can definitively be correlated with oxidative stress has yet to be established.

Prebiotics, probiotics, and synbiotics have the potential to modulate the gut microbiota, which has been linked to mood and mental health. Prebiotics are non-digestible fibres that promote the growth of beneficial gut bacteria, while probiotics are live microorganisms that confer health benefits when consumed in adequate amounts. Synbiotics combine prebiotics and probiotics to enhance their effects synergistically. Several studies have suggested that these interventions may have antidepressant effects by influencing neurotransmitter pathways, reducing inflammation, and improving gut barrier function (reviewed in [125]). However, the evidence is still evolving, and more robust clinical trials are needed to determine their efficacy, optimal dosages, and long-term effects. Additionally, individual responses to prebiotics, probiotics, and synbiotics can vary, highlighting the importance of personalised treatment approaches in psychiatric care.

## 7. Role of Nutraceuticals in Modulating Response to Medication

Nutraceuticals are widely utilised by patients with depression and are often self-prescribed [126]. A nutraceutical product may be defined as a substance or product, which has physiological benefits or provides protection against chronic disease [127]. Of those with depression, over half will have tried nutraceuticals, either as an alternate to taking medication or as an adjunct for those already prescribed medication [128]. To address the issue of medication non-response in depression, the use of nutraceuticals as an adjunct to antidepressants have been suggested as a potential way of augmenting response. These agents generally have favourable side effect profiles and have the potential to act on brain pathways associated with depression [129,130]. In fact, numerous studies have shown that nutrients are a valuable adjunct to standard medication in depression [128,129,130,131]. A recent meta-analysis found evidence which supports that the supplementation of nutrients, including omega-3, vitamin D, methyl-folate, and S-adenosylmethionine (SAMe), with antidepressants helps to reduce the symptoms of depression [130]. Table 2 below highlights their proposed mechanism of action in depression and in augmentation of response to antidepressant medications.

Regarding supplementation in depression, it is noteworthy to consider the impact of nutraceuticals on the one-carbon cycle and the methylation process. The one carbon cycle refers to a metabolic pathway involved in the endogenous synthesis of the monoamine neurotransmitters serotonin, dopamine, and noradrenaline (norepinephrine), in which one-carbon groups are transferred between several intracellular substrates. Dysfunction in this pathway may contribute to the development of the symptoms of depression [133]. Both SAMe and methyl-folate are components in the one carbon cycle and act as methyl donors for monoamine neurotransmitter synthesis [133,134,135]. Although they are endogenously produced from micronutrient substrates, they can be deficient for various physiological reasons, including in the case of methyl-folate and *MTHFR* genetic polymorphisms [136]. Thus, deficiency in either compound has been linked to depression and supplementation has been shown to improve depression symptoms as well as responsiveness to antidepressant medications. 

Along with their role in methylation, both SAMe and methyl-folate have been suggested to reduce oxidative stress and inflammation [134]. This points to the antioxidant role of these nutrients, as with other nutrients, implicated in the potential treatment of depression. As previously mentioned, studies have shown that the ‘oxidative–antioxidant’ function of the body in patients with depression is dysfunctional, mainly manifested in the increased concentration of oxygen free radicals and the abnormal activity of some antioxidant enzymes; namely, copper–zinc superoxide dismutase (CuZn SOD), glutathione peroxidase (GPX), and catalase (CAT) [137]. Thus, the role of antioxidant nutraceuticals seems important in managing oxidative stress in depression and may augment response to medication. 

Furthermore, of the biomarkers implicated in predicting medication response in depression, the urinary pyrrole test as well as the zinc/copper ratio have been shown to be modulated by nutraceuticals [83,110]. For elevated pyrrole levels, the addition of vitamin B6 and zinc has been traditionally prescribed to reduce this level [83], although our recent study had suggested that it is the antioxidant effect of zinc, and not vitamin B6, that reduces urinary pyrroles, as it is well understood that vitamin B6 does not bind to pyrroles due to the incompatibility of the relevant chemistries [82]. According to clinicians in this field of nutritional psychiatry, ongoing treatment with high-dose vitamin B6 and zinc is required to reduce or suppress symptoms of mental illness [84,85]. However, this has not been without harm, as numerous cases of B6 toxicity have been recorded, which again highlights the need for better understanding of the biochemistry involved [138]. It is possible that there is a potential benefit of B6 treatment in mental health illness, which may be due to its antioxidant properties. However, further research is needed to identify other antioxidants, such as N-acetyl cysteine, that may be equally or more effective without the side-effects of accumulation [139]. 

As we have clinically observed, iron deficiency anaemia may also result in elevated urinary pyrroles. It is proposed that when iron levels are low, the body upregulates HPL as an alternative mechanism to bind and prevent the loss of free haemoglobin and thus iron loss is associated with the breakdown of haemoglobin. Therefore, iron deficiency needs to be excluded when interpreting elevated urinary pyrrole levels. 

For the zinc/copper ratio, the addition of zinc reduces elevated copper, due to the reciprocal relationship between these two minerals [110]. According to a recent cohort study, individuals with the highest zinc consumption were about 30–50% less likely to suffer from depression than individuals with the lowest zinc consumption [140]. Studies have also indicated that the use of zinc supplementation in conjunction with antidepressant medications may increase drug efficacy. Interestingly, this effect does not appear to be mediated through the impact of zinc on inflammatory processes, suggesting that other factors are at play [141]. 

## 8. Conclusions and Recommendations

Currently, no single biomarker or group of biomarkers has been successfully integrated into clinical practice for diagnosing depression or as the guiding choice of medication decisions. The complex nature of depression poses significant challenges for biomarker discovery, but it also offers the potential to identify multiple underlying biomarkers that define specific subgroups. The identification of potential biomarkers will facilitate personalised depression treatment, leading to quicker and more effective therapeutic interventions. Furthermore, the role of nutraceuticals in modifying one’s response to antidepressant medications needs further investigation to determine whether correlations can be drawn between nutraceutical use and potential intrinsic biomarkers of depression. To date, studies have largely investigated the role of nutraceuticals in depression management as isolative nutrients rather than in combination. Nutraceuticals often work as complement and tandem cofactors in intrinsic biochemical pathways and may benefit those who do not respond to traditional medications. Ideally, further work could focus on investigating the role of combinations of synergistic nutraceuticals rather than individual nutrients. Additionally, investigating whether specific SNPs identified through PGx testing influence the response to adjunctive nutraceuticals is a critical area for exploration. At this point in time, we recommend clinicians cautiously consider multi-gene PGx testing, especially in cases of medication resistant depression and especially for drugs that have recommended indications. The combination of pharmacogenomic testing in conjunction with existing clinical decision-making tools provides the treating physician with additional guidance in therapeutic planning. However, it does not discount the complex nature of depression nor other factors that could impact therapeutic outcome. Overall, while PGx testing holds promise for optimising depression treatment, its widespread implementation requires further evidence of cost-effectiveness, use in diverse population where PGx data are limited, and integration into standard clinical practice guidelines.

## Figures and Tables

**Table 1:** CYP450 enzymes and the antidepressants they metabolise (focus on major metabolising enzymes) [48,49]. Note that not all these drugs have pharmacogenomic indications. * indicates minor metabolising pathway.

PGx	Medication Substrate
CYP2D6	AMITRIPTYLINE
	FLUOXETINE
	FLUVOXAMINE
	DULOXETINE
	MIRTAZAPINE
	PAROXETINE
	VENLAFAXINE (pro-drugs)
	VORTIOXETINE
CYP2C19	AMITRIPTYLINE
	CITALOPRAM
	DESVENLAFAXINE
	ESCITALOPRAM
	SERTRALINE
CYP3A4	DESVENLAFAXINE *
	VENLAFAXINE * (pro-drug)
CYP1A2	AGOMELATINE

**Table 2:** Nutraceuticals and their role in depression management [130,131,132].

Nutraceutical	Mechanism of Action	Evidence of Efficacy
Omega-3 fatty acids	Anti-inflammatory properties; Targeting of lipid rafts and G coupled protein receptors influences neurotransmission	Some evidence suggests benefits in combination with antidepressants
S-Adenosylmethionine (SAMe)	Precursor for monoamine neurotransmitters; Involved in methylation processes; Anti-inflammatory effects	Possible efficacy as an adjunctive medication to antidepressants
Methyl-folate	Improved monoamine neurotransmitter synthesis and activity; Anti-inflammatory effects; Restoring intrinsic SAMe levels	May enhance response to antidepressant medications
Vitamin D	Modulation of neurotransmitters, Serotonin synthesis, Anti-inflammatory effects	Modulation of neurotransmitters, Serotonin synthesis, Anti-inflammatory effects
Iron	Norepinephrine regulation; regulation of BDNF levels	Evidence suggests benefits in those that are iron deficient
Zinc	Involved in neurotransmission, antioxidant properties	Limited evidence for efficacy as an adjunct in depression
